# A multi-omic analysis to investigate the causal associations between circulating proteins and risk of spontaneous abortion and their potential implications

**DOI:** 10.5935/1518-0557.20250031

**Published:** 2025

**Authors:** Min Huang, Meihua He, Jiahui Xiang, Yinghui Liu, Limei Zhang, Xiaoli Sun

**Affiliations:** 1 Affiliated Hospital and Medical School of Nantong University, Jiangsu, China; 2 Center for Reproductive Medicine, Affiliated Hospital of Nantong University, Nantong University, Jiangsu, China

**Keywords:** spontaneous abortion risk, genetic variants, multi-omic analysis, Mendelian randomization, causal associations

## Abstract

**Objective:**

Spontaneous abortion is a complex disorder with a significant genetic component. Identifying genetic variants influencing spontaneous abortion risk could unveil biological pathways and potential therapeutic targets.

**Methods:**

We performed Mendelian randomization using cis- and trans-protein quantitative trait loci (pQTLs) as instrumental variables to assess causal effects of circulating proteins on spontaneous abortion. Proteins exhibiting differential expression between sexes were excluded. KEGG pathway enrichment was employed to investigate the pathways affected by susceptibility genes, while single-cell transcriptomic analysis was utilized to explore the susceptible cell types with elevated expression of these genes within the uterine endometrium.

**Results:**

MMP9 and DC-SIGN were associated with increased spontaneous abortion risk (OR=1.11(1.03-1.19), P=3.70x10_-3_; OR=1.09(1.02-1.16), *p*=9.89x10_-3_), while HBAZ and NELL1 had protective effects (OR=0.96(0.94- 0.99), *p*=5.20x10_-3_; OR=0.94(0.9-0.98), *p*=8.54x10_-3_). Additionally, TMM85 conferred higher spontaneous abortion risk (OR=1.06(1.02-1.1), *p*=4.72x10_-3_). Pathway analysis highlighted sphingolipid binding, chemorepellent activity, and tumor necrosis factor receptor activity. Single-cell transcriptomics revealed that MUL1, EMC4, NDC80, and SELL genes exhibit higher expression levels within uterus cells, and these susceptibility genes displayed elevated expression levels in leukocytes, mature NK T cells, and T cells in the uterus.

**Conclusions:**

Our integrated multi-omics analysis identified genetic variants influencing spontaneous abortion risk and their downstream molecular mechanisms, providing insights into potential therapeutic targets. The implicated pathways and cell types may guide future investigations into the pathogenesis of spontaneous abortion.

## INTRODUCTION

Spontaneous abortion, commonly referred to as miscarriage, is defined as the unintentional termination of a pregnancy before the 20th week of gestation. It represents a significant proportion of pregnancy complications, with estimates suggesting it occurs in 10-15% of clinically recognized pregnancies. This number may be even higher if very early miscarriages are taken into account. The causes of spontaneous abortion are multifaceted and complex. Maternal factors such as age, health conditions like diabetes or thyroid disorders, and lifestyle factors like smoking and alcohol use can contribute to the risk of miscarriage (Yuan *et al.*, 2021). Fetal chromosomal abnormalities are another significant cause, often resulting from errors in cell division that lead to abnormalities in the number or structure of chromosomes. Uterine abnormalities, including fibroids or an unusually shaped uterus, can also interfere with the implantation or development of the embryo. Accumulating evidence has underlined the influence of genetic factors in spontaneous abortion (de Ziegler *et al.*, 2016). Recent advancements in genetic research, particularly genome-wide association studies (GWAS), have shed light on various genetic loci associated with an increased risk of miscarriage (Laisk *et al.*, 2020). These studies scan the genomes of many individuals to find genetic markers that can be associated with a particular disease or condition. Through GWAS, researchers have been able to identify specific genetic variants that are more common in women who have experienced spontaneous abortion, suggesting a genetic component to this condition. Family- based studies further support the role of genetics in spontaneous abortion. Evidence suggests that women with a first-degree relative who has experienced a miscarriage have a 2- to 4-fold increased risk of experiencing a miscarriage themselves. This familial aggregation of spontaneous abortion cases indicates a potential hereditary component.

Despite these advances, the genetic architecture underlying spontaneous abortion is not fully understood. Elucidating this complex network of genetic factors could provide critical insights into the biological basis of spontaneous abortion. Understanding the genetic predispositions and mechanisms could guide the development of predictive tools, preventative strategies, and therapeutic interventions. This could help reduce the incidence of spontaneous abortion and support women and families affected by this challenging condition. Proteins represent key effector molecules situated downstream of genetic variation that mediate phenotypic manifestation. Consequently, investigating proteins influenced by genetic variants that also impact spontaneous abortion risk can help characterize putative causal pathways. Although earlier candidate gene studies have evaluated select protein biomarkers, unbiased profiling of the plasma proteome enabled by mass spectrometry now allows examination of the proteome-wide influence of genetic variation. Specifically, protein quantitative trait loci (pQTLs) that associate genomic variants with plasma protein levels provide instruments to probe the causal role of proteins in spontaneous abortion via Mendelian randomization.

The integration of multiple layers of omics data is paramount in interpreting the broader biological context of potentially causative proteins. Various types of omics data, such as genomics, transcriptomics, proteomics, and metabolomics, offer a holistic view of the complex biological systems. Among these, enrichment analysis can play a crucial role by connecting implicated proteins to relevant pathways and cellular processes, which may uncover the underlying mechanisms of disease pathogenesis or biological phenomena, not only provides a detailed snapshot of the biological system at play but also opens avenues for further research and discovery.

Therefore, in this study, we sought to delineate genetic variants influencing spontaneous abortion susceptibility and elucidate their downstream molecular consequences by analyzing multi-omic datasets encompassing genetic variation, plasma proteomic profiles, and single-cell transcriptomes. By integrating pQTL data from a large-scale study of the plasma proteome with spontaneous abortion outcomes from population biobanks and single-cell RNA-sequencing data from the uterus, this multi-pronged approach can provide unique insights into the genomic architecture and causal pathways governing spontaneous abortion risk.

## MATERIALS AND METHODS

### DataSource

1,927 pQTLs associated with 1,478 proteins, including 589 cis-pQTLs and 1,391 trans-pQTLs were extracted from the work of Sun *et al.* (2018), which investigated the genetic architecture of the human plasma proteome. This study identified numerous genetic associations with 1,478 plasma proteins and unveiled their connections to gene expression, disease-associated loci, and potential therapeutic targets.

Information regarding the outcomes of spontaneous abortions was obtained from Finngen (Kurki *et al.*, 2023) (R9_O15_ABORT_SPONTAN), including data from 16,906 cases and 149,622 controls. This dataset offers insights into habitual abortion, specifically defined as three or more consecutive spontaneous abortions, with a focus on the endpoint “O15_ABORT_SPONTAN.” It encompasses phenotype data for 473,681 individuals, which narrows down to 265,735 females after applying sex-specific criteria. Following genotype quality control filtering, 20,775 females were retained, forming this dataset after excluding ‘O15_PREG_ABORT.’ The data is sourced from hospital discharge and cause of death records, with exclusions based on specific endpoints. Additionally, the dataset provides mortality risk estimates for females of varying ages with this condition, offering valuable insights into its long-term implications. We want to acknowledge the participants and investigators of the FinnGen study.

Single-cell sequencing information was sourced from The Tabula Sapiens Consortium (2022). The Tabula Sapiens project represents a comprehensive molecular reference atlas comprising over 400 human cell types. The consortium utilized single-cell transcriptomics to analyze mRNA molecules in nearly 500,000 cells across 24 tissues and organs. This extensive dataset offers novel insights into the utilization of the human genome to generate diverse cell types within the human body. We extracted expression data from 16 of these cell types, specifically 10,500 cells from the uterus.

### Data Control

Since spontaneous abortion only occurs in females, our study’s first step involved excluding proteins with significant associations (*p*<1x10_-5_) between protein levels and sex. This step was taken to ensure the robustness of downstream analyses and to prevent errors introduced by proteins that exhibit differential expression between different genders.

### Mendelian Randomization

Mendelian randomization is a statistical method aimed at understanding causal relationships between potential risk factors and disease outcomes based on observational data. It aims to overcome the challenges of conducting randomized trials, which are often impractical, unethical, or expensive. The method addresses the issue of correlation without causation, common in observational studies, by using genetic variants as instrumental variables. These genetic variants, linked to risk factors, are less susceptible to confounding and reverse causation because they are established at birth and inherited independently. Therefore, any associations observed in a Mendelian randomization study are more likely to be causal, providing a powerful tool for epidemiological analysis.

### Mendelian Randomization with cis-pQTLs

For proteins with cis-pQTLs, these cis-pQTLs were utilized as instrumental variables (IVs) in Two-sample mendelian randomization analyses to investigate their potential causal relationship with spontaneous abortion. For proteins with only one cis-pQTL, the Wald ratio was the primary method employed. In cases where two or more cis-pQTLs were present, the Inverse Variance Weighted method was used.

The Wald ratio, a straightforward technique, is employed when a singular genetic variant, also known as an instrumental variable (IV), is available. The ratio is calculated as the quotient of the effect of the genetic variant on the outcome (e.g., disease susceptibility) to its impact on the exposure (e.g., protein levels), operating under the assumption that the genetic variant influences the outcome solely through the exposure. Despite the Wald ratio’s simplicity and ease of interpretation, its reliability may be compromised when the genetic variant has a weak effect on the exposure or when multiple genetic variants are associated with the exposure. Conversely, the IVW method is applicable when multiple genetic variants are present. It calculates the causal effect estimate as the weighted mean of the Wald ratios, with weights corresponding to the inverse of the variance of the Wald ratios. This method assumes the validity of all genetic variants (i.e., they impact the outcome exclusively through the exposure) and the absence of pleiotropy (i.e., genetic variants do not influence the outcome via other pathways). While the IVW method offers a more precise and reliable estimate of the causal effect than the Wald ratio when these assumptions hold, it can be biased if these assumptions are violated, such as in the presence of pleiotropy. This analysis was conducted using TwoSampleMR 0.5.7 in R 4.1.3.

### Mendelian Randomization with trans-pQTLs

For proteins that lacked cis-pQTLs, our study employed trans-pQTLs as IVs to assess their impact on the risk of spontaneous abortion. The trans-pQTLs underwent pruning for linkage disequilibrium, with LD r2 <0.001 within 10,000kb windows. For proteins with only one trans-pQTL, the Wald ratio was the primary method used. In cases where two or more trans-pQTLs were present, the Inverse Variance Weighted method was employed. This analysis was also conducted using TwoSampleMR 0.5.7 in R 4.1.3.

### Result Aggregation and Validation

Due to multiple testing, this study defined significant associations as those with *p* < 0.01. Results that exhibited significant associations with spontaneous abortion were aggregated. For proteins with two or more instrumental variables, we utilized the mr-egger method to examine the presence of horizontal pleiotropy. Additionally, we conducted a search for significant SNP-trait associations (*p*<5×10_−8_) documented in the PhenoScanner database.

In this study, a *p*-value threshold of 0.01 was used to account for multiple testing and reduce the likelihood of false positives.

### KEGG Enrichment Analysis

For proteins significantly associated with spontaneous abortion, genes encoding these target proteins were subjected to KEGG pathway enrichment analysis, providing insights into potential functional pathways. This analysis was performed using clusterProfiler 4.2.2 in R 4.1.3.

### Single-Cell Transcriptomics Analysis

Our study leveraged single-cell transcriptomes from 16 distinct cellular contexts, comprising 10,500 cells from the Uterus, to annotate and analyze the expression profiles of genes encoding target proteins. We extracted their expression levels across these 16 cell types based on proteins significantly associated with spontaneous abortion to identify susceptible cells within the Uterus. This analysis was conducted using cellxgene.census 1.6.0 in R 4.1.3.

## RESULTS

### Data Control

Detailed information on 1,927 pQTLs associated with 1,478 proteins, comprising 589 cis-pQTLs and 1,391 transpQTLs, can be found in the supplementary materials. Additionally, following the exclusion of proteins exhibiting significant associations (*p*<0.00001) between protein levels and sex, there were 500 proteins with cis-pQTLs and 400 proteins without cis-pQTLs but with 400 trans-pQTLs. The specific information regarding these proteins and pQTLs can also be found in the supplementary materials.

### Mendelian Randomization with cis-pQTLs

Regarding proteins with cis-pQTLs, our results indicate that MMP-9 and DC-SIGN are associated with an increased risk of spontaneous abortion (OR=1.11(1.03-1.19), *p*=0.0037; OR=1.09(1.02-1.16), *p*=0.00989, while HBAZ and NELL1 exhibit a protective effect against spontaneous abortion (OR=0.96(0.94-0.99), *p*=0.0052; OR=0.94(0.9- 0.98), *p*=0.00854.

### Mendelian Randomization with trans-pQTLs

For proteins lacking cis-pQTLs, and thus utilizing transpQTLs as instrumental variables (IVs), TMM85 was found to be associated with an increased risk of spontaneous abortion (OR=1.06(1.02-1.1), *p*=0.00472.

### Result Aggregation and Validation

In the context of multiple testing, no significant association results were found for MMP-9 (rs2250889) in PhenoScanner (*p*<1e-5). However, it’s worth noting that DCSIGN, HBAZ, NELL1, and TMM85’s instrumental variables all showed associations with other traits. Furthermore, the introduction of horizontal pleiotropy by trans-pQTLs was found to be much greater than that by cis-pQTLs. The details of associations with IVs can be found in the supplementary materials.

### KEGG Enrichment Analysis

**Figure 1:**
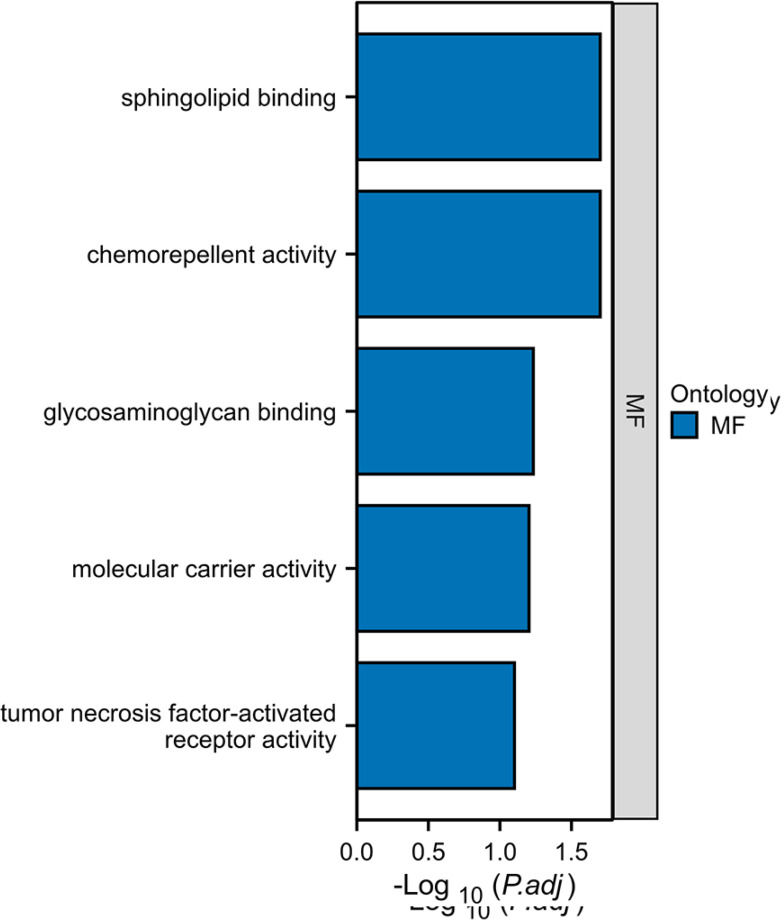
Our study conducted KEGG pathway enrichment analysis for the genes associated with spontaneous abortion at *p*<0.05 in MR results, including ENTPD1, CD209, FLRT2, GLRX2, GLTPD2, HBZ, MYORG, LMNB1, LILRA4, MMP9, NELL1, SEMA3G, SELL, TMEM132C, TNFRSF1B, TNFAIP6, EMC4, NDC80, PRRG1, MUL1).

Our study conducted KEGG pathway enrichment analysis for the genes associated with spontaneous abortion at *p*<0.05 in MR results, including ENTPD1, CD209, FLRT2, GLRX2, GLTPD2, HBZ, MYORG, LMNB1, LILRA4, MMP9, NELL1, SEMA3G, SELL, TMEM132C, TNFRSF1B, TNFAIP6, EMC4, NDC80, PRRG1, MUL1), as shown in Figure 1. The analysis revealed significant associations between these genes and pathways related to sphingolipid binding, chemorepellent activity, glycosaminoglycan binding, molecular carrier activity, and tumor necrosis factor-activated receptor activity. The details of susceptible genes can be found in the supplementary materials.

### Single-Cell Transcriptomics Analysis

**Figure 2:**
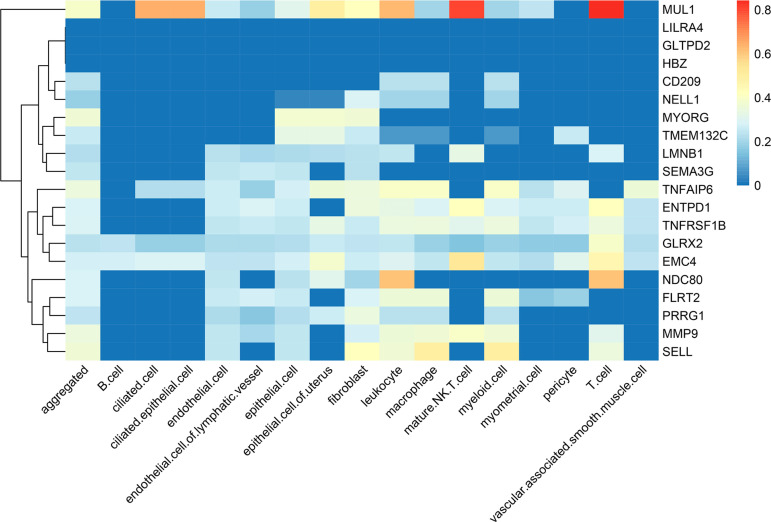
The expression profiles of these 20 susceptibility genes across 16 different types of uterine endometrial cells.

We examined the expression profiles of these 20 susceptibility genes across 16 different types of uterine endometrial cells, as depicted in Figure 2. Our findings revealed that MUL1, EMC4, NDC80, and SELL exhibit higher expression levels within uterus cells. Furthermore, these 20 susceptibility genes also displayed elevated expression levels in leukocytes, mature NK T cells, and T cells.

## DISCUSSION

In summary, this study utilized a multi-omic approach to investigate the genetic underpinnings and molecular mechanisms associated with spontaneous abortion risk. By integrating proteomic, genomic, and single-cell transcriptomic data, we identified key genetic variants influencing spontaneous abortion susceptibility and their downstream effects. Our Mendelian randomization analysis revealed that MMP9 and DC-SIGN were associated with increased spontaneous abortion risk, while HBAZ and NELL1 demonstrated a protective effect. Additionally, TMM85 was also found to confer increased spontaneous abortion risk. Pathway analysis showed significant involvement of sphingolipid binding, chemorepellent activity, and tumor necrosis factor receptor activity. Single-cell transcriptomics revealed that MUL1, EMC4, NDC80, and SELL exhibit higher expression levels within uterus cells. These findings provide valuable insights into the genetic architecture and potential therapeutic targets for spontaneous abortion, and suggest directions for future research into its pathogenesis.

Degradation and remodeling of the extracellular matrix (ECM) is a vital event in all processes involved in normal human reproduction (Hulboy *et al.*, 1997). The enzymes matrix metalloproteinases (MMPs), particularly MMP9, primarily enable this remodeling of the ECM (Cabral-Pacheco *et al.*, 2020). This is essential for endometrial decidualization, as well as trophoblast implantation and placentation. Matrix metalloproteinase-9 (MMP-9), also known as gelatinase B, is a type of enzyme that belongs to the matrix metalloproteinase (MMP) family (Yu *et al.*, 2019). These enzymes are known for their ability to degrade components of the extracellular matrix, which is the non-cellular component present within all tissues and organs. MMP-9 specifically has the ability to break down type IV and type V collagen, which are key components of the basement membrane of the extracellular matrix. This makes MMP- 9 particularly important in processes such as embryonic development, reproduction, tissue remodeling, and disease processes including inflammation and cancer (Yan *et al.*, 2021). In the context of disease, MMP-9 is often implicated in processes that involve tissue remodeling or cell migration, such as in tumor metastasis. High levels of MMP-9 have been found in various types of cancers, and it is thought to contribute to the spread of cancer cells by breaking down the extracellular matrix, allowing the cells to invade surrounding tissues. MMP9 plays a crucial role in the final differentiation of human endometrial stromal cells into decidual cells (Fisher, 2004; Seval *et al.*, 2004). As these enzymes are found in decidual tissues throughout pregnancy, they are considered critical regulators of trophoblast invasion and angiogenesis (Niu *et al.*, 2000; Husslein *et al.*, 2009). Research has suggested that functional gene polymorphisms of MMP9 -1562 C/T might be associated with an increased risk of Idiopathic recurrent spontaneous abortion in women (Pereza *et al.*, 2012). Moreover, a rise in MMP-9 concentrations has been linked to spontaneous abortion (Castruita-De la Rosa *et al.*, 2020).

This study demonstrates several notable strengths, including the implementation of a comprehensive multi-omic strategy that integrates genetic, proteomic, and single-cell transcriptomic data. This robust approach facilitates an indepth investigation into the genetic risk factors associated with spontaneous abortion, thereby enabling the elucidation of causal pathways and potentially viable therapeutic targets. The large sample size, encompassing data from 16,906 cases and 149,622 controls, significantly enhances the statistical power and lends credibility to the study’s findings. Furthermore, the application of Mendelian randomization bolsters the causal inference drawn between the identified genetic variants and the risk of spontaneous abortion, thereby circumventing common pitfalls in observational studies such as confounding and reverse causation. The innovative assimilation of data from diverse sources, including population biobanks and single-cell transcriptomics, provides a unique and comprehensive insight into the genetic architecture and causal pathways underpinning spontaneous abortion risk.

However, it is essential to acknowledge a few limitations. First, the study’s analysis was predominantly based on populations of European ancestry, which may restrict the extrapolation of findings to other ethnic groups. Second, the reliance on existing databases and available datasets may introduce potential biases that could skew the results. Lastly, although Mendelian randomization significantly reduces the likelihood of confounding, it cannot entirely eliminate it. Consequently, the interpretation of the study’s results should be undertaken with this caveat in mind.

## CONCLUSION

In conclusion, this comprehensive multi-omic study has provided novel insights into the genetic architecture and potential therapeutic targets for spontaneous abortion. Through the application of Mendelian randomization, we identified key genetic variants influencing spontaneous abortion susceptibility and their downstream molecular mechanisms. Notably, our findings indicated an increased spontaneous abortion risk associated with MMP9, DC-SIGN and TMM85, while a protective effect involving HBAZ and NELL1. Furthermore, through KEGG pathway analysis and single-cell transcriptomics, we highlighted significant involvement of pathways such as sphingolipid binding, chemorepellent activity, and tumor necrosis factor receptor activity, and revealed elevated expression of susceptibility genes in uterine cells. Despite the study limitations, including potential biases from the reliance on existing databases and the predominance of data from populations of European ancestry, our findings offer a valuable resource for future investigations into the pathogenesis of spontaneous abortion. Future research efforts are warranted to validate these findings in diverse populations and to further explore the identified pathways and gene targets for potential therapeutic interventions.
